# A Pilot Study of Younger Sibling Adaptation: Contributions of Individual Variables, Daily Stress, Interparental Conflict and Older Sibling’s Variables

**DOI:** 10.5964/ejop.2139

**Published:** 2021-05-31

**Authors:** Laura Merino, Ana Martínez-Pampliega, David Herrero-Fernández

**Affiliations:** a Social and Developmental Psychology Department, Psychology and Education Faculty, University of Deusto, Bilbao, Spain; b Health Sciences Faculty, European University of the Atlantic, Santander, Spain; The Ohio State University Wexner Medical Center, Columbus, OH, USA

**Keywords:** sibling relations, interparental conflict, temperament, sibling dyads, child siblings

## Abstract

Older siblings are powerful socialization agents, playing a significant role in the sociocognitive, social, and emotional development of their younger siblings. However, there are few clues about the variables that explain younger sibling’s adaptation. The objective of this pilot study was to identify the determinants of younger siblings' adaptation and to analyze the role played by personal, sibling, family and older siblings’ variables using 50 dyads of siblings aged between 7 and 18 years. The variables considered were the sibling relationships and the maladaptation of older siblings, and individual (sex, number of siblings, extroversion, and agreeableness) and contextual variables (interparental conflict, daily stress) were controlled. Hierarchical multiple regressions provided evidence in favor of the model that analyzed the younger siblings' maladaptation to school, showing positive associations both with the older siblings' level of school maladaptation and with sibling conflict. In addition, the study highlighted the relevance of the trait of agreeableness and of family stress in the adaptation of younger siblings.

Siblings spend a lot of time together every day and this shared time has become an important scenario of socialization (Kumar, Thomas, & Deb, 2015; Updegraff, McHale, Whiteman, Thayer, & Delgado, 2005) in different cultures (Buist et al., 2014). In the 1980s, numerous works began to describe the influence of sibling relations on the social and emotional development of children and adolescents (Menesini, Camodeca, & Nocentini, 2010). Since then, the literature has highlighted that relationships among siblings are relevant for the comprehension of children’s sociocognitive (Dunn, Brown, Slomkowski, Tesla, & Youngblade, 1991), social (Kramer & Bank, 2005; McElwain & Volling, 2005) and emotional development (Brown & Dunn, 1996), and therefore, siblings can be considered powerful socialization agents. This is reflected when the children highlight their sense of collectivity and of feeling part of a group, as well as protection and emotional security as the greatest contributions of their siblings in their lives (Edwards, Mauthner, & Hadfield, 2005).

Therefore, developmental psychologists have analyzed the contributions of sibling relations to the process of socialization of children and adolescents (Brody, 1998; Dunn, Slomkowski, & Beardsall, 1994; Kramer & Conger, 2009; Kumar et al., 2015). In addition, these sibling relations have an important impact on children’s psychological well-being (McHale, Updegraff, & Whiteman, 2012; Ponappa, Bartle-Haring, Holowacz, & Ferriby, 2017) and increasingly more results appear that underscore this (Conger & Kramer, 2010; Kennedy & Kramer, 2008).

Concerning ways for the older siblings' socialization of the younger one, an extensive literature has shown that social learning and modeling are key processes through which older siblings influence the behavior and adaptation of their younger siblings (Bandura, 2001; Whiteman, Becerra Bernard, & McHale, 2010). One of the most supported perspectives to explain the mechanisms associated with some behavioral problems and the relationship between siblings is Bandura’s (1973) social learning theory, applied specifically to this field through the work carried out by Patterson (1984), and Dunn and Munn (1986).

Following this line, various studies have examined the role of big brother/sister as a significant model in the socialization of the other siblings in different areas, both positive and negative: alcohol and drug use (Fagan & Najman, 2005; Slomkowski, Rende, Novak, Lloyd-Richardson, & Niaura, 2005; Trim, Leuthe, & Chassin, 2006; Tsamparli & Frrokaj, 2016); gambling (Canale et al., 2017); sexual behavior and teen pregnancies (East & Jacobson, 2001; McHale, Bissell, & Kim, 2009; Patterson, 1984; Rodgers, Rowe, & Harris, 1992; Slomkowski et al., 2005; Wheeler et al., 2016); risky health behavior (D’Amico & Fromme, 1997); externalizing and internalizing behaviors (Branje, Van Lieshout, Van Aken, & Haselager, 2004); criminal offences (Slomkowski, Rende, Conger, Simons, & Conger, 2001); attitudes and activities according to gender role (Crouter, Whiteman, McHale, & Osgood, 2007; McHale, Updegraff, Helms-Erikson, & Crouter, 2001); intimacy and control in friendship relations (Updegraff, McHale, & Crouter, 2002); academic and leisure interests (Jensen & McHale, 2015; Whiteman, McHale, & Crouter, 2007), and development of empathy (Tucker, Updegraff, McHale, & Crouter, 1999) and social skills (Stormshak et al., 1996).

Specifically, older siblings can be a significant authority to learn how to succeed in the area of friends, particularly in environments outside the home, such as school or neighborhood (Zukow-Goldring, 2002). Although there are few works that analyze the function of siblings in academic performance, there is evidence that highlights the role of the siblings as possible significant models for academic success (Bouchey, Shoulberg, Jodl, & Eccles, 2010; Jensen, Pond, & Padilla-Walker, 2015).

## Transfer to Other Socialization Contexts

Finally, some researchers postulate that sibling relations are a training stage for social skills where children and adolescents acquire interpersonal strategies that they also transfer to adaptive behaviors in other contexts, such as their friendship relations (Buhrmester, 1992; Menesini et al., 2010). Regardless of the quality of the sibling relations, the stage shared by the siblings is a privileged social context where they receive and give affection, establish interactions of play, and put into practice negotiation and conflict-resolution skills (Dunn et al., 1994), as well as prosocial behaviors (Pike & Oliver, 2017) that will serve them in their peer relations outside of the family. Some studies reaffirm the idea that there is a strong association between sibling relations and friendship relations in young adults (MacKinnon-Lewis, Starnes, Volling, & Johnson, 1997; Stormshak et al., 1996).

## Other Relevant Variables in Adaptation: Family Socialization Context and Individual Variables

Regarding family variables that affect individual adaptation, numerous investigations have revealed a significant relationship between interparental conflict and child adaptation difficulties (Buchanan & Heiges, 2001; Davies, Martin, & Cicchetti, 2012; Morgado Camacho & González Rodríguez, 2001; Dillman Taylor, Purswell, Lindo, Jayne, & Fernando, 2011), highlighting internalizing (Hornor, 2005) and externalizing symptoms (Formoso, Gonzales, & Aiken, 2000) and problems with children’s social relations (e.g., sibling and peer relationships; Martínez-Pampliega et al., 2015).

Everyday family stressors can also lead to children's poorer adaptation (Crnic & Greenberg, 1990). For example, it has been shown that the daily problems derived from child-raising, difficulties to combine work and family life, and marital conflicts have a negative impact on families and children (Crnic, Gaze, & Hoffman, 2005; Crnic & Greenberg, 1990; Crouter, Bumpus, Head, & McHale, 2001; Lee, Vernon‐Feagans, Vazquez, & Kolak, 2003). In this sense, there is much evidence of the influence of family stressors on children's concern about their family relationships and, therefore, their involvement in them (cf. for a review, see Cummings, Davies, & Simpson, 1994). Children whose family must deal with stressors that are maintained over time show greater sensitization to stress, which increases their reaction, concern, and efforts to exert some control and power in the situation of family distress (Nixon & Cummings, 1999).

Regarding individual variables that affect individual adaptation, many personality traits predict the individual's social competences. For instance, more extroverted and agreeable children are socially more competent over time, whereas children with high levels of negative emotionality and low levels of frustration have more social problems such as aggressive behaviors, anger, anxiety, and avoidance (Asendorpf & Van Aken, 2003).

## Aim of the Study

Therefore, the objective of this pilot study is to identify the determinants of younger siblings' adaptation and to analyze the role played by personal, sibling, family and older sibling’s variables. For this purpose, we intended to differentiate the contributions of sex, personality, age, number of siblings, experienced daily stress, perceived interparental conflict, sibling relationships, and the older sibling’s individual variables (personal, social, school, and family maladaptation) to explain the younger sibling’s adaptation.

## Method

### Participants

The sample was made up of 50 pairs of siblings relatively close in age within dyads, with age ranging between 7 and 18 years across dyads, mean age 11.9 years (*SD* = 2.81). The mean age of the older siblings was 13.3 years (*SD* = 2.30) and the mean age of the younger siblings was 10.5 years (*SD* = 2.30). On the one hand, 28 older siblings were boy, whereas other 22 older siblings were girls. On the other hand, 22 younger siblings were boys, whereas 28 younger siblings were girls. The families of these pairs of siblings had an average of 2.3 children (*SD* = 0.58). The sample had the same number of boys and girls, 50, respectively.

We used a sample that was restricted to the population of students of the historical territory of Bizkaia (Autonomous Community of the Basque Country). The sample of participants was made up of pairs of siblings from different public and private schools. The inclusion criteria for participation in this study were: being a student of between 3rd grade of Primary School and 2nd grade of High School of the Spanish educational system; the participants should be biological or adoptive siblings (despite the fact that there were no adoptive siblings). The exclusion criterion was having a lot of difficulty to understand the questionnaires. The inclusion and exclusion criteria were formulated a priori.

Regarding the parents' marital status, most were married in first nuptials (67%), followed by divorced couples (27%), and the remaining couples were either married in second nuptials (4%) or living with a partner (2%). About 60% of the fathers and mothers were middle or highly qualified professionals (fathers = 59.6%, mothers = 58%), whereas the greatest differences between fathers and mothers were found in the number of unskilled workers (fathers = 14.9%, mothers = 36%), executives (fathers = 8.5%, mothers = 0%), workers in small businesses (fathers = 12.8%, mothers = 6%), and unemployed workers (fathers = 4.3%, mothers = 2%). Nevertheless, there was no significant differences on the working situation between fathers and mothers (χ^2^ *=* 20.418, *p* = .432).

### Procedure

To implement the assessment protocol, we contacted the school principals and counselors of various schools of Bizkaia, according to the researcher's convenience, but attempting to achieve representativeness in terms of source of financing (public and concerted schools) and location (different neighborhoods and cities). We attended to all the families from all these schools that were interested in participating in the study and that met the inclusion criteria.

When contacting the schools, we explained the general goal of the study, emphasizing that the school's collaboration only consisted of passing on the information about the study to the families. The participation of the families in the study required that two of their children complete questionnaires. The parents who were interested in the participation of two of their children subsequently contacted the researcher via email or phone. In this first contact with the families, we explained the main purpose of the study, ensuring confidentiality and anonymity of the data, and that we would give 5 euros to each sibling for their cooperation. Each family chose the most convenient date and time for them to participate in the study. They could participate in a classroom of their children's school or on the premises of DeustoPsych (Psychological laboratory of the University of Deusto). The parents of the participants signed an informed consent. Both siblings answered all the questionnaires in a room where a research assistant was available to solve any doubts and support the youngest participants with the questionnaire fulfillment.

### Instruments

#### Sibling Relationship

##### Sibling Relationships Questionnaire (SRQ)

This child self-report, SRQ (Furman & Buhrmester, 1985), assesses the quality of sibling relationship through 48 items (e.g., “How often do you and your sibling go places or do things together?"), of which the first 42 items are rated on a 5-point Likert type scale ranging from 1 (*Hardly at all*) to 5 (*Very much*). The exception are 6 items about maternal and paternal partiality, which assess favoritism, attention, and differential treatment of siblings by the mother and the father on a 5-point Likert scale ranging from -2 (*almost always favors/pays attention to/treats my sib better than me)* to 2 (*almost always favors/pays attention to/treats me better*). The SRQ has 16 scales (Prosocial, Similarity, Intimacy, Companionship, Admiration by Sibling, Admiration of Sibling, Affection, Nurturance by Sibling, Nurturance of Sibling, Dominance by Sibling, Dominance of Sibling, Antagonism, Competition, Quarreling, Maternal and Paternal Partiality) whose score is calculated by summing the mean score of the 3 items of each scale. In turn, the scales are grouped into four main dimensions of sibling relationships (Warmth/Closeness, Sibling Power/Status, Conflict and Maternal/Paternal Partiality) that are the ones used in this study. Each main dimension is calculated by adding its scales, except for the dimension of *Sibling Power/Status* which, by recommendation of the original authors of the SRQ (Furman & Buhrmester, 1985), is calculated by subtracting passive from active scores: (*Nurturance by Sibling* + *Dominance by Sibling*) - (*Nurturance of Sibling* + *Dominance of Sibling*). There were no validated questionnaires in Spanish to assess sibling relationships, so the English version of SRQ was translated, adapted and validated to Spanish in another study (author, unpublished manuscript), and it replicates the original structure. Validity information for the Sibling Relationships Questionnaire can be obtained from the main author of this manuscript. In this study, the reliabilities of the four main factors were: Warmth/Closeness α = .88, Sibling Power/Status α = .83, Conflict α = .76, and Partiality α = .56.

#### Family Variables

##### Children's Perception of Interparental Conflict Scale (CPIC-Y)

We used the Spanish version adapted from the original scale (McDonald & Grych, 2006), which replicates the structure of the three main scales although the items are grouped in different subscales (author, unpublished manuscript). This self-report for children and adolescents contains 22 dichotomous items (1 = *Yes*, 0 = *No*) grouped into three scales: (1) Conflict Properties: this includes 11 items distributed in three subscales, with 6 items about children's perception of Negative Interparental Conflict, 3 items about their perception of Constructive Interparental Conflict and 2 items about their perception of Aggression in Interparental Conflict (e.g., “ I've seen or heard my father and my mother arguing”); (2) Threat Scale: the second scale has 6 items referring to the children's feelings of threat and fear in the face of interparental conflict (e.g., “ I get scared when my father and my mother argue”); (3) and Self-Blame: the third scale includes 4 items related to the children's feelings of self-blame for interparental conflict (e.g., “ When my father and my mother argue, it is usually my fault”). The reliability indices (McDonald´s ω, as the items were dichotomous) in this study were: Conflict Properties ω = .82, Threat ω = .81, Self-Blame ω = .48.

##### Inventario Infantil de Estresores Cotidianos [Child Inventory of Everyday Stressors] (IIEC)

This 41-item inventory (School and Peer and Family dimensions), which presents everyday stressors selected by experts as representative of the construct of daily stress (Trianes Torres et al., 2009), is rated by the children (1 = *Yes*, 0 =*No*). The Cronbach alpha internal consistency of the test is .70 and the test-retest reliability reaches a score of .78 (Trianes Torres et al., 2009). Only the items relating to the area of School and Peers (School and social stress: 12 items) and Family (Family stress: 17 items) were used in this study. The reliability index (McDonald´s omega, as the items were dichotomous) in this study for the dimension of School and Social stress was ω = .49 and for Family stress, it was ω = .61.

#### Personality Traits

##### Big Five Questionnaire-Children and Adolescents (Dimensions of Extroversion and Agreeableness)

For this study, we only evaluated two dimensions that are most closely linked to interpersonal relationships: Extroversion and Agreeableness (Barbaranelli, Caprara, Rabasca, & Pastorelli, 2003). These dimensions contain 13 and 12 items, respectively, which describe the frequency of occurrence of certain extroversion and agreeableness traits. Items are rated on a 5-point Likert scale ranging from 1 (*hardly ever*) to 5 (*almost always*). This instrument has solid international psychometric support (Barbaranelli, Caprara, & Rabasca, 2006), where Extraversion reached a Cronbach alpha of .71 and Agreeableness reached a Cronbach alpha of .80. However, we used the Spanish version adapted by Ortiz, Tello, and del Barrio Gándara (2005) and, in this study, Cronbach's alpha was .66 for Extroversion and .84 for Agreeableness.

#### Child Adaptation

##### Test Autoevaluativo Multifactorial de Adaptación Infantil [Multifactorial Child Adaptation Self-Assessment Test] (TAMAI)

This questionnaire (Hernández, 1983) evaluates children's general, personal, school, and social maladaptation, and dissatisfaction with family and siblings through 175 dichotomous items (1 = *Yes*, 0 = *No*) answered directly by the children. Children’s maladaptation is described as the appearance of difficulties and problems in the contexts where children participate (persona, social, school and family). In this study, we used the scales of general, personal, school, and social maladaptation and family dissatisfaction: (1) The Personal Maladaptation scale has 39 items and encompasses both self-maladaptation and maladaptation to manage daily issues or a personal difficulty to accept reality; it had a McDonald´s omega reliability of .84; (2) The School Maladaptation scale includes 31 items assessing poor learning performance and disruptive behavior in the classroom; it obtained a McDonald´s omega of .81; (3) The Social Maladaptation scale describes the degree of difficulty in social relationships through 35 items; it had a McDonald´s omega of .78. These three scales are grouped into a single dimension called General Maladaptation; (4) The Family Dissatisfaction scale includes 5 items that indicate the degree of dissatisfaction with the climate of the home and the interparental relationship; its McDonald´s omega reliability was .60. In this study, the internal reliabilities of the dimensions were adequate (General Maladaptation ω = .91, Personal Maladaptation ω = .84, School Maladaptation ω = .81, Social Maladaptation ω = .78, Family Dissatisfaction ω = .60).

### Analysis Strategy

First, descriptive statistics (mean, standard deviation, and range) of all the variables were calculated. After verifying the normality of variables, we calculated the bivariate correlation matrix (Pearson's *r*) among the main variables of the study. Only young siblings’ data were included to calculate the descriptive statistics and the correlations. Lastly, we conducted a hierarchical multiple regression to analyze the variables that explain a greater percentage of the variance of the younger siblings' adaptive behavior. Demographics (sex, age, number of siblings in the family) and temperament variables (extraversion and agreeableness) were introduced in the first block; in the second step, we included family variables (threat, self-blame, and properties of interparental conflict; school and social stress and family stress); the third step included sibling relations (warmth, conflict, power, and partiality); and the last step included the variable of the older sibling's maladjustment corresponding to the dependent variable in each model (e.g., if the dependent variable is the younger sibling's school maladjustment, in this fourth step, we included the variable of the older sibling's school maladjustment). Statistical power was calculated for each one of the five regression models with G*Power (Version 3.1.9.4) software (Faul, Erdfelder, Lang, & Buchner, 2007).

## Results

### Descriptive Analysis

Table 1 provides means, standard deviations, and correlations for key study variables based on the data of younger siblings. Regarding the individual variables, significant associations emerged, especially between the trait of agreeableness and the level of individual maladjustment in the total score, and in social, family, and school maladaptation, with significant, negative and small or moderate correlations. With regard to family variables, the data showed that both the properties of interparental conflict and the feeling of self-blame for it and daily social and school stress were significantly and positively associated with different levels of maladaptation. Finally, regarding the sibling variables, sibling warmth was significantly and negatively associated with all levels of maladaptation, except for the level of personal maladaptation. However, sibling conflict was significantly and positively associated with the younger sibling's total and school maladaptation scores.

**Table 1:** Descriptive Statistics and Correlations Between Variables of the Data From Younger Siblings

Variable	*M*	*SD*	Range	2	3	4	5	6	7	8	9	10	11	12	13	14	15	16	17	18
1. Age	10.40	2.33	7 - 16	.16	.16	−.08	−.55**	−.08	.13	−.07	.10	.22	−.13	.35*	.05	−.03	.06	−.18	−.17	.08
2. Number of siblings	2.32	0.59	2 - 4		−.17	−.12	−.03	−.06	.20	.22	.53**	−.02	−.12	−.16	.32*	.04	.22	−.26	−.04	.22
3. Extroversion	44.64	6.07	27 - 57			.63**	.04	.20	.02	−.09	−.05	.61**	.08	.26	.18	−.23	−.26	.04	−.13	−.29*
4. Agreeableness	52.33	7.97	32 - 68				.07	−.01	−.20	−.21	.05	.54**	.23	−.14	.15	−.40**	−.38**	−.08	−.25	−.43**
5. Threat	3.48	2.09	0 - 6					.12	.16	−.05	.15	−.23	.18	−.13	.23	.17	.06	.26	.22	.11
6. Self-blame	0.48	0.91	0 - 4						.07	.07	−.07	−.02	.18	.11	.20	.27	.10	.48**	.23	.05
7. Conflict properties	2.86	2.53	0 - 11							.32*	.19	−.29	.22	.11	.21	.34*	.23	.20	.55**	.36*
8. School and social stress	1.58	1.30	0 - 5								.05	−.34*	.07	.03	−.03	.31*	.44**	.05	.08	.28
9. Family stress	1.31	1.42	0 - 6									−.07	−.16	−.08	.10	−.05	.08	−.12	.11	−.02
10. Sibling warmth	76.72	11.38	52 - 105										−.19	.07	.05	−.41**	−.42**	−.20	−.33*	−.34*
11. Sibling conflict	26.08	6.71	10 - 40											.02	.16	.35*	.11	.37*	.27	.32*
12. Sibling power	−5.92	5.57	−16 - 5												.09	.09	.01	.11	−.04	.07
13. Parental partiality	0.46	2.31	−8 - 8													.14	.16	.09	−.15	.16
14. General maladaptation of younger sibling	27.02	12.02	5 - 60														.83**	.80**	.45**	.79**
15. Social maladaptation of younger sibling	7.96	4.38	0 - 23															.51**	.28	.55**
16. Personal maladaptation of younger sibling	9.39	5.50	0 - 22																.41**	.36*
17. Family dissatisfaction of younger sibling	0.56	0.93	0 - 3																	.38**
18. School maladaptation of younger sibling	9.18	5.03	1 - 19																	-

*p < .05. **p < .001.

### Associations Between Maladaptation and Dissatisfaction of Younger and Older Siblings

Table 2 shows the correlations between general, social, personal and school maladaptation and family dissatisfaction of younger and older siblings. Only two correlations are significant: family dissatisfaction of younger sibling has a significant and positive association with the family dissatisfaction of older sibling; and younger sibling’s school maladaptation has a significant and positive association with the older sibling’s school maladaptation.

**Table 2:** Correlations Between Maladaptation Variables of Younger and Older Siblings

Variable	General maladaptation of older sibling	Social maladaptation of older sibling	Personal maladaptation of older sibling	Family dissatisfaction of older sibling	School maladaptation of older sibling
General maladaptation of younger sibling	.19	.19	.15	.11	.18
Social maladaptation of younger sibling	.24	.26	.26	.21	.10
Personal maladaptation of younger sibling	−.01	.04	.11	.19	−.08
Family dissatisfaction of younger sibling	.07	−.01	−.08	.37*	.23
School maladaptation of younger sibling	.24	.18	.04	.12	.37*

*p < .05.

### Predicting Young Siblings’ Adaptation

Table 3 reports results from five hierarchical multiple regression models that predicted younger siblings’ maladaptation from individual, family, and sibling variables. As it can be observed, all of the models attained high statistical power (1-β), with values ranging from 87.4% to 99.4%. Only the model that explains the younger sibling's school maladjustment was significant, accounting for 68% of the variance. Specifically, this model had four variables that yielded a significant association with the younger sibling's school maladjustment: sex was negatively associated, and family stress, sibling conflict, and the older sibling's level of school maladjustment were positively associated. The other models were not significant to explain the younger sibling's maladaptation but two variables—younger sibling's sex (negative correlation) and family stress (positive correlations)—were significantly associated with the younger sibling's maladaptation. Lastly, the younger sibling's family maladjustment was also significantly and negatively associated with the number of siblings in the family and with sibling partiality.

**Table 3:** Hierarchical Multiple Regression Analysis to Predict Younger Sibling Adaptation (N = 50)

Variable	General maladaptation of younger sibling (1-β = 99.4%)			Social maladaptation of younger sibling (1-β = 87.4%)			Personal maladaptation of younger sibling (1-β = 98.8%)			Family dissatisfaction of younger sibling (1-β = 99.4%)			School maladaptation of younger sibling (1-β = 98.5%)		
	B^a^	*SE* B^a^	β^a^	B^b^	*SE* B^b^	β^b^	B^c^	*SE* B^c^	β^c^	B^d^	*SE* B^d^	β^d^	B^e^	*SE* B^e^	β^e^
Age	1.47	1.43	.28	0.01	0.50	.01	0.38	0.66	.16	−0.09	0.07	−.22	0.81	0.46	.38^†^
Gender (1 = boy, 2 = girl)	−8.88	4.38	−.37^†^	−1.18	1.64	−.13	−1.43	2.01	−.13	−0.48	0.25	−.27^†^	−4.58	1.61	−.45*
Number of siblings	0.03	4.06	.01	1.36	1.61	.15	−2.833	2.06	−.27	−0.71	0.29	−.38*	1.49	1.56	.15
Extroversion	−0.10	0.59	−.06	−0.05	0.23	−.07	0.10	0.27	.13	−0.02	0.04	−.12	−0.26	0.23	−.34
Agreeableness	−0.52	0.43	−.37	−0.15	0.17	−.28	−0.14	0.21	−.22	−0.03	0.03	−.28	−0.21	0.16	−.35
Threat	0.80	1.58	.13	−0.45	0.57	−.21	0.58	0.68	.21	−0.01	0.09	−.03	0.54	0.52	.22
Self-blame	0.34	2.98	.03	−0.68	0.83	−.15	1.98	1.24	.35	0.16	0.13	.18	−0.25	0.82	−.05
Conflict properties	0.40	1.81	.06	0.28	0.56	.11	−0.61	0.80	−.20	0.10	0.11	.19	0.40	0.55	.14
School and social stress	0.75	1.85	.09	0.39	0.69	.11	0.07	0.88	.02	0.01	0.11	.01	0.30	0.62	.08
Family stress	3.43	1.61	.43*	1.33	0.63	.42*	1.60	0.76	.44*	0.27	0.10	.42*	0.86	0.60	.25
Sibling warmth	−0.02	0.27	−.02	−0.09	0.11	−.24	−0.05	0.12	−.12	0.02	0.02	.24	0.14	0.10	.32
Sibling conflict	0.69	0.33	.41	0.08	0.12	.13	0.16	0.15	.21	0.01	0.02	.02	0.32	0.12	.45*
Sibling power	−0.05	0.38	−.02	0.04	0.15	.05	−0.08	0.18	−.08	−0.01	0.03	−.08	0.17	0.15	.18
Maternal/Paternal partiality	0.48	1.04	.08	0.43	0.42	.18	0.24	0.51	.09	−0.15	0.07	−.30*	0.09	0.41	.03
General maladaptation of older sibling	0.08	0.21	.08												
Social maladaptation of older sibling				0.07	0.19	.07									
Personal maladaptation of older sibling							−0.03	0.25	−.03						
Family dissatisfaction of older sibling										0.26	0.15	.30			
School maladaptation of older sibling													0.38	0.17	.37*

^a^*R*^2^ = .71, *p* > .05. ^b^*R*^2^ = .58. *p* > .05. ^c^*R*^2^ = .69. *p* > .05. ^d^*R*^2^ = .71. *p* > .05. ^e^*R*^2^ = .68. *p* = .034.

*p < .05. ^†^.10 > *p* > .05.

## Discussion

This study drew on two perspectives: a perspective based on the findings that confirm that sibling relations have an important impact on children's psychological well-being (McHale et al., 2012); the other perspective focused on the theory of social learning, which emphasizes modeling as a key process whereby older siblings influence the behavior and adaptation of their younger siblings (Bandura, 2001; Whiteman et al., 2010). Therefore, the objective of this study was to analyze the variables that explain younger siblings' adaptive behavior, differentiating the contributions of variables of the sibling relationship and variables of the older siblings, as well as taking into account other family and individual variables.

On the one hand, the correlational analyses of this study reveal the protector role of the trait of agreeableness against the younger siblings' level of maladaptation, confirming that certain temperament traits like this are associated with children's higher levels of social adaptation (Asendorpf & Van Aken, 2003). Following with the possible protective factors against child maladjustment, the negative correlation between sibling warmth and various levels of maladaptation in this study supports the positive and close relations between siblings as a facilitator of children's adaptation, as already indicated in other studies (Conger & Kramer, 2010; Kennedy & Kramer, 2008). This result supports continuing to analyze the processes described in the buffering hypothesis that posits that positive sibling relationships are associated with children’s lower levels of internalizing symptoms (Pike, Coldwell, & Dunn, 2005; Richmond, Stocker, & Rienks, 2005). Finally, the associations between different aspects of interparental conflict and everyday stress and the level of child maladjustment are not surprising, given the extensive literature that identifies both variables as clearly harmful for children and adolescents' health and adaptation (Crnic & Greenberg, 1990; Crnic et al., 2005; Crouter et al., 2001).

Notwithstanding the foregoing, the model that analyzed the younger siblings' maladaptation to school was significant, showing positive associations both with the older siblings' level of school maladaptation and with sibling conflict. According to these results, there is also a significant and positive association between the school maladaptation of both siblings. Therefore, these results could be interpreted from the classical theory of social learning (Bandura, 2001) of siblings’ mutual influence, because the association of both siblings' level of school maladaptation can be considered as an evidence of the correlation between siblings’ academic outcomes. In line with this, future studies with causal design could study the role of older siblings as models in the academic area for their younger siblings, supporting the scarce literature on the subject (Bouchey et al., 2010). The sibling relations are an important scenario of socialization due to the great amount of time they spend together through the whole lifespan and because siblings are usual the first same-age individual that a person interact with. From this perspective, the association between sibling conflict and the younger siblings' school maladjustment can be considered evidence of the association of sibling relations, in this case negative, on the children's health and well-being (Conger & Kramer, 2010; McHale et al., 2012).

In addition, this model emphasizes other variables that explain younger siblings' school adaptation: sex, in particular being female, protects against school maladjustment (Kaur & Chawla, 2016). Some authors have explained results like this because girls have better social and emotional adjustment, which is associated with better academic adjustment (Kasinath, 2003). Moreover, in this study family stress appears to be a factor that favors younger siblings' school maladjustment (Crnic & Greenberg, 1990).

Finally, we note several limitations of this study. The sample size is not negligible, given that we used sibling dyads of children and adolescents. Therefore, the sample size does not allow obtaining definitive conclusions based just on these results and this research line needs to enlarge the sample to attain more demonstrative results. Increasing the sample size of future studies would allow the separate analysis of sibling relations and socialization processes in childhood and adolescence, as well as improve the statistical power of the analyses. Moreover, the age range of the sample is too broad (from 7 to 18 years-old participants) and that could affect the results because there are differences in the sibling relationships, individual adaptation and family processes depending on the developmental stage of the sons and daughters. Also, some of the subscales of the instruments do not reach adequate validity values, which hinders the interpretation of the results of this study. An additional limitation is that this study used an unpublished Spanish translated assessment of sibling quality. Future studies would benefit from longitudinal designs that would allow making causal inferences to explain younger siblings' adaptation. Finally, the results of this study are limited to Basque families due to the places where the sample was obtained, so there is a need to replicate studies in this area with different samples, especially with participants from non-Western cultures to analyze the impact of cultural differences in the adaptation of younger siblings. Therefore, taking into account all the mentioned limitations, this research line would need future studies with a longitudinal methodology compared to this pilot study, in order to produce causal conclusions.
